# Aortic Valve Endocarditis with Anomalous Origin of the Right Coronary Artery and Unknown Infected Thrombus in the Dissected Descending Thoracic Aorta

**DOI:** 10.1055/s-0040-1714715

**Published:** 2020-11-05

**Authors:** Juan Caceres, Vikram Sood, Linda Farhat, Bo Yang

**Affiliations:** 1 University of Michigan Medical School, University of Michigan, Ann Arbor, Michigan; 2 Department of Cardiac Surgery, University of Michigan, Ann Arbor, Michigan

**Keywords:** aortic dissection, anomalous right coronary artery, endocarditis

## Abstract

We report an intricate aortic root replacement in a young male patient suffering from native valve infective endocarditis due to *Serratia marcescens* . Further complicating the total root replacement, there was an unknown infected aortic thrombus and a concomitant anomalous right coronary artery with an intramural course. As a result of our more aggressive approach, we believe that we lowered the risk of recurrent infection of the bioprosthesis of the aortic root.

## Introduction

*Serratia marcescens* is an uncommon causative agent in native valve infective endocarditis. 1 Coronary anomalies, specifically anomalous coronary arteries with intramural courses, increase the risk of morbidity and mortality associated with surgical treatment of infective endocarditis. It is also unclear how to manage a thrombus in a dissected proximal descending thoracic aorta in the setting of acute infective endocarditis.

## Case Presentation

**Fig. 1:**
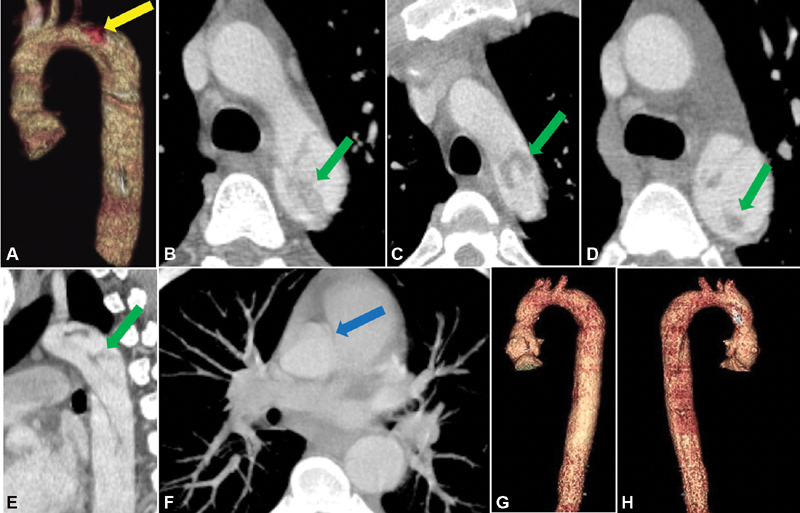
Preoperative computed tomography (CT) images: ( **A** ) CT anterior reconstruction of thoracic aorta with thrombus (yellow arrow). ( **B–D** ) Axial and ( **E** ) sagittal CT images of filling defects caused by dissection and thrombus (green arrows). ( **F** ) Axial image of the anomalous coronary artery (blue arrow). Postoperative CT images: ( **G** ) anterior and ( **H** ) posterior reconstructions of thoracic aorta.

A 24-year-old man with a past medical history of bicuspid aortic valve and intravenous (IV) drug use was admitted from an outside facility for management of native aortic valve infective endocarditis secondary to *S. marcescens* likely secondary to the patient's IV drug use. A transesophageal echocardiogram (TEE) demonstrated a thickened bicuspid aortic valve with moderate aortic stenosis (mean gradient: 33 mm Hg) and severe insufficiency. A computed tomography (CT) scan revealed a chronic Stanford's Type-B aortic dissection originating just distal to the left subclavian artery, luminal thrombus within the distal aortic arch and proximal descending thoracic aorta ( Fig. 1A–E ), splenic infarcts, and bilateral renal infarcts. The patient was placed on the appropriate intravenous antibiotics and started on systemic anticoagulation.

**Fig. 2:**
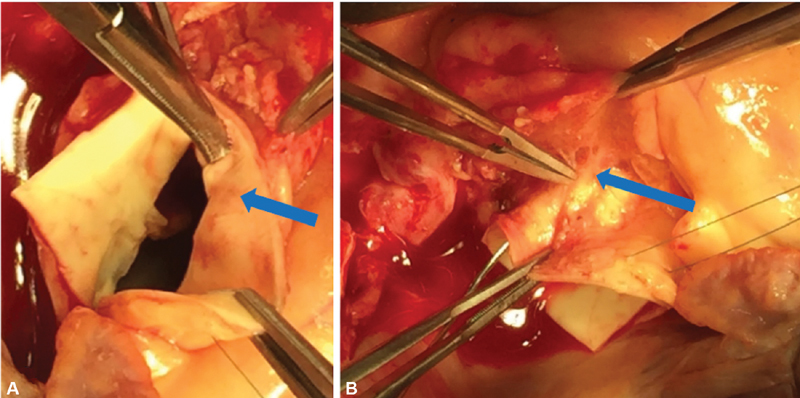
Anomalous right coronary artery. ( **A** ) Intramural course (arrow). ( **B** ) Right coronary artery outside the aortic root (arrow).

After 2 weeks of persistently negative blood cultures, the patient was taken to the operating room. An 8 mm Hemashield graft was sewn to the innominate artery and used for arterial inflow. The aorta was divided just above the sinotubular junction, and the aortic root was examined. The valve was grossly infected. There was a root abscess and tissue necrosis that extended from the right-noncommissural post to the left–right interleaflet triangle through the right coronary sinus. After extensive debridement, it was clear that the cavities were very large and continuous, thus warranting total aortic root replacement. The left coronary artery button originated from the left coronary sinus. Upon further examination of the right coronary artery, it was noted to originate from the left coronary sinus and travel in an intramural course toward the right coronary sinus ( Fig. 2A ). The tunnel portion measured from 7 to 10 mm. As such, the proximal intraluminal portion of the right coronary was unroofed to the origin of the right coronary artery proper within the right coronary sinus. Carefully, the right coronary artery was dissected out from the outside of the aortic root, and the right coronary button was trimmed around the true ostium of the right coronary artery ( Fig. 2B ).

Circulatory arrest was initiated at 24°C with unilateral anterograde cerebral perfusion via the innominate artery. The aorta was incised longitudinally from the ascending aorta to the distal arch. There was a chronic Type-B dissection that extended to the distal descending thoracic aorta. The initial intimal tear was distal to the left subclavian artery. Upon looking down the distal arch and proximal descending aorta, a large amount of thrombus was attached to the dissection flap, but not to the aortic wall, located within the proximal descending aorta. The flap, measuring approximately 3 cm wide and 6- to 8-cm long was completely removed along with the thrombus. The arch was back bled to flush any remaining debris, the longitudinal aortotomy was closed, and circulation was resumed.

A total root replacement was then completed with a 29-mm freestyle aortic valve (Medtronic). The unroofed right coronary button was anastomosed in an end-to-side fashion, taking great care to preserve the thin outer layer of the tunnel. Finally, the aortic arch was mobilized to meet the freestyle root without an interposition graft. This anastomosis was augmented anteriorly with a patch of noninfected aortic root tissue from the noncoronary sinus. The cardiopulmonary bypass time was 294 minutes and the cross-clamp time was 253 minutes.

The patient recovered well and was discharged with 6 weeks of IV antibiotics. He was in normal sinus rhythm and did not necessitate postoperative permanent pacemaker implantation. Interestingly, his aortic arch thrombus, aortic valve leaflets, and aortic root tissue all grew *S* . *marcescens* . At 3 months, the surface echocardiogram demonstrated a normal functioning aortic valve with a mean gradient of 7 mm Hg. There was no aortic valve or paravalvar regurgitation and no signs of bioprosthetic aortic valve endocarditis. A postoperative CT demonstrated no intraluminal thrombus ( Fig. 1G and H ). The patient has had no recurrence of infection for 2 years since surgery.

## Discussion

This case report describes an intricate aortic root debridement and replacement in a patient suffering from native valve infective endocarditis due to *S* . *marcescens* with infected thrombus, which was unknown before surgery. There are three key points in this case as follows: (1) extensive damage of the aortic root can be caused by *S* . *marcescens* that could not be detected preoperatively by routine imaging modalities, (2) anomalous right coronary artery reimplantation as a button can be done after unroofing the intramural course, (3) aortic thrombus in infective endocarditis further complicates the management of infective endocarditis.

The *S* . *marcescens* bacteremia, commonly a nosocomial infection, was likely due to the patient's IV drug use. He was likely bacteremic 2 weeks prior to admission when he started developing infectious symptoms which gave the bacteria plenty of time to proliferate in various organs, including his bicuspid aortic valve. He also had a history of bicuspid aortic valve with stenosis which could have facilitated bacterial attachment to tissue due to turbulence. Upon arrival to our hospital, the patient was stabilized and given the appropriate antibiotics. There were no conduction abnormalities noted preoperatively. Interestingly, the preoperative echocardiogram and CT only identified the vegetation on the aortic valve and were unable to define the extensive necrosis of the surrounding tissue around the aortic root. Given the patient's young age and lack of risk factors for coronary artery disease, a coronary angiogram or CT scan was not performed. Due to the patient's extensive necrosis within the aortic root, total aortic root replacement was required. The operation was further complicated by the incidental, intraoperative finding of an anomalous origin, and intramural course of the right coronary which could not be well appreciated on the preoperative CT ( Figs. 1F and 2 ). The right coronary artery required unroofing and further dissection from outside the aortic root for additional accommodation. In similar cases, anomalous coronary anatomy has been approached with coronary artery bypass grafting (CABG) rather than coronary unroofing. 2 While CABG eliminates the need to anatomically modify the aorta, it can predispose the patient to need for future interventions on the bypass graft. Despite the higher risk for intraoperative complications associated with coronary unroofing, this procedure effectively eliminates the need for future intervention on a bypass graft. Coronary unroofing, initially reported by Mustafa et al, 3 provides a potentially long-lasting method for the correction of the anomalous and intramural coronary artery, particularly in young patients.

Contrary to common practice, which would suggest definitive anticoagulation as treatment for the luminal thrombus which was unknown to be infected, we chose to remove the luminal thrombus within the aorta. This tissue subsequently cultured positive for *S* . *marcescens* , demonstrating the importance of its removal for the prevention of subsequent infections. As a result of our more aggressive approach to the luminal aortic thrombus, we believe that we lowered the risk of recurrent infection of bioprosthesis of the aortic root. We also tried to avoid addition of any foreign material to replace the ascending aorta.

In conclusion, this case report demonstrates management of a patient suffering from *S* . *marcescens* aortic valve infective endocarditis. Our case suggests that the safest management of a necrotic aortic root is total aortic root replacement. Furthermore, when presented with an anomalous and intramural right coronary artery within a necrotic aortic root, one may choose to unroof the coronary artery rather than perform coronary artery bypass, with safe results. Finally, in patients with infective endocarditis like ours, surgical resection of intraluminal thrombi may be the most effective therapeutic strategy for long-term protection of the bioprosthetic valve.
